# New mini- zincin structures provide a minimal scaffold for members of this metallopeptidase superfamily

**DOI:** 10.1186/1471-2105-15-1

**Published:** 2014-01-03

**Authors:** Christine B Trame, Yuanyuan Chang, Herbert L Axelrod, Ruth Y Eberhardt, Penelope Coggill, Marco Punta, Neil D Rawlings

**Affiliations:** 1 Joint Center for Structural Genomics, La Jolla, CA, 92037, USA; 2 Stanford Synchrotron Radiation Lightsource, SLAC National Accelerator Laboratory, Menlo Park, CA, 94025, USA; 3 Sandford-Burnham Institute, La Jolla, CA, 92037, USA; 4 Wellcome Trust Sanger Institute, Hinxton, Cambridgeshire, CB10 1SA, UK; 5 European Molecular Biology Laboratory, European Bioinformatics Institute, Hinxton, Cambridgeshire, CB10 1SD, UK

**Keywords:** Acel_2062, Metallopeptidase, Zincin, JCSG, Structural genomics

## Abstract

**Background:**

The Acel_2062 protein from *Acidothermus cellulolyticus* is a protein of unknown function. Initial sequence analysis predicted that it was a metallopeptidase from the presence of a motif conserved amongst the Asp-zincins, which are peptidases that contain a single, catalytic zinc ion ligated by the histidines and aspartic acid within the motif (**H**EXX**H**XXGXX**D**). The Acel_2062 protein was chosen by the Joint Center for Structural Genomics for crystal structure determination to explore novel protein sequence space and structure-based function annotation.

**Results:**

The crystal structure confirmed that the Acel_2062 protein consisted of a single, zincin-like metallopeptidase-like domain. The Met-turn, a structural feature thought to be important for a Met-zincin because it stabilizes the active site, is absent, and its stabilizing role may have been conferred to the C-terminal Tyr113. In our crystallographic model there are two molecules in the asymmetric unit and from size-exclusion chromatography, the protein dimerizes in solution. A water molecule is present in the putative zinc-binding site in one monomer, which is replaced by one of two observed conformations of His95 in the other.

**Conclusions:**

The Acel_2062 protein is structurally related to the zincins. It contains the minimum structural features of a member of this protein superfamily, and can be described as a “mini- zincin”. There is a striking parallel with the structure of a mini-Glu-zincin, which represents the minimum structure of a Glu-zincin (a metallopeptidase in which the third zinc ligand is a glutamic acid). Rather than being an ancestral state, phylogenetic analysis suggests that the mini-zincins are derived from larger proteins.

## Background

A metallopeptidase is a proteolytic enzyme that has one or two metal ions as an integral part of its catalytic machinery located within the active site. There are many families of metallopeptidases that bind a single zinc ion required for catalysis. The zinc ion is tetrahedrally co-ordinated by three residues from the peptidase and a water molecule that becomes activated to be the nucleophile in the catalytic reaction. In many of these families, but not all, the residues that ligate the zinc ion (referred to here as “ligands”) are two histidines within an HEXXH motif, and a third coordinating residue that is C-terminal to this motif, which can be a glutamic acid, a histidine or an aspartic acid. Metallopeptidases with an HEXXH motif are known as zincins. In a metallopeptidase such as thermolysin, the third zinc ligand is usually a glutamic acid, and these peptidases are known as Glu-zincins. In a metallopeptidase such as matrix metallopeptidase 1 (MMP1), the zinc ligands are the three histidines within an HEXXHXXGXXH motif. In MMP1 there is also an important region known as the Met-turn, in which there is a conserved methionine that structurally supports the active site. For this reason, metallopeptidases such as MMP1 are known as Met-zincins [1]. In some zincins the third zinc ligand may be an aspartic acid (within the motif HEXXHXXGXXD), and there is no Met-turn; these peptidases are known as Asp-zincins [2]. All of the zincins share structural similarities and in the MEROPS classification and database, the different families that can be recognized by sequence similarities are all included in clan MA. This clan is subdivided into three subclans. Subclan MA(E) containing the Glu-zincins, subclan MA(M) containing the Met-zincins and subclan MA(D) containing the Asp-zincins [3].

A zincin structure contains at least two subdomains: an N-terminal subdomain which includes the HEXXH motif, and a C-terminal subdomain that includes the third zinc ligand. The active site is therefore between the two subdomains. Within the zincins, the size of the C-terminal subdomain varies enormously, from being large in the matrix metallopeptidases (peptidase family M10), to being just a single helix, which is the case for snapalysin from *Streptomyces lividans* (peptidase family M7), which has one of the smallest sequences and structures in the clan [4].

Very recently, a minimal structure for a Glu-zincin has been determined [5], representing the smallest known member of this subclan so far discovered. No catalytic activity could be detected. The family has been provisionally assigned the name M95, but will not appear in the MEROPS database until peptidase activity has been experimentally confirmed. Lenart *et al.*[6] identified a number of protein families in which an HEXXH motif was conserved. One of these families was Pfam family PF06262 (DUF1025)[Pfam:PF06262], which includes atleast 400 sequences from bacteria. Members of this family have the Asp-zincin-like motif HEXXHXXGXXD. In this paper, we report the structure of a member of this family: the Acel_2062 protein from *Acidothermus cellulolyticus*, a cellulolytic thermophile found in hot springs [7]. The domain architecture represents a minimal structure for a zincin, with very little sequence beyond the third zinc ligand and an absence of the Met-turn.

## Methods

### Protein expression and purification

The American Type Culture Collection (ATCC) provided the genomic DNA used to clone *Acel_2062* (ATCC Number: ATCC 43068). Protein production and crystallization of the Acel_2062 protein was carried out by standard JCSG protocols [8]. Clones were generated using the Polymerase Incomplete Primer Extension (PIPE) cloning method [9]. The gene encoding *Acel_2062* (GenBank: YP_873820[GenBank:YP_873820]; UniProtKB: A0LWM4[UniProtKB:A0LWM4]) was synthesized with codons optimized for *Escherichia coli* expression (Codon Devices, Cambridge, MA) and cloned into plasmid pSpeedET, which encodes an expression and purification tag followed by a tobacco etch virus (TEV) protease cleavage site (MGSDKIHHHHHHENLYFQ/G) at the amino terminus of the full-length protein. *Escherichia coli* GeneHogs (Invitrogen) competent cells were transformed and dispensed on selective LB-agar plates. The cloning junctions were confirmed by DNA sequencing. Expression was performed in a selenomethionine-containing medium at 37°C. Selenomethionine was incorporated via inhibition of methionine biosynthesis [10], which does not require a methionine auxotrophic strain. At the end of fermentation, lysozyme was added to the culture to a final concentration of 250 μg/ml, and the cells were harvested and frozen. After one freeze/thaw cycle the cells were homogenized in lysis buffer [50 mM HEPES, 50 mM NaCl, 10 mM imidazole, 1 mM Tris(2-carboxyethyl)phosphine-HCl (TCEP), pH 8.0] and passed through a Microfluidizer (Microfluidics). The lysate was clarified by centrifugation at 32,500 x g for 30 minutes and loaded onto a nickel-chelating resin (GE Healthcare) pre-equilibrated with lysis buffer, the resin was washed with wash buffer [50 mM HEPES, 300 mM NaCl, 40 mM imidazole, 10% (v/v) glycerol, 1 mM TCEP, pH 8.0], and the protein was eluted with elution buffer [20 mM HEPES, 300 mM imidazole, 10% (v/v) glycerol, 1 mM TCEP, pH 8.0]. The eluate was buffer exchanged with TEV buffer [20 mM HEPES, 200 mM NaCl, 40 mM imidazole, 1 mM TCEP, pH 8.0] using a PD-10 column (GE Healthcare), and incubated with 1 mg of TEV protease per 15 mg of eluted protein for 2 hours at 20°–25°C followed by overnight at 4°C. The protease-treated eluate was passed over nickel-chelating resin (GE Healthcare) pre-equilibrated with HEPES crystallization buffer [20 mM HEPES, 200 mM NaCl, 40 mM imidazole, 1 mM TCEP, pH 8.0] and the resin was washed with the same buffer. The flow-through and wash fractions were combined and concentrated to 15.6 mg/ml by centrifugal ultrafiltration (Millipore) for crystallization trials.

### Protein crystallization

The Acel_2062 protein was crystallized using the nanodroplet vapor diffusion method [11] with standard JCSG crystallization protocols [12]. Sitting drops composed of 100 nl protein solution mixed with 100 nl crystallization solution in a sitting drop format were equilibrated against a 50 μl reservoir at 277 K for 72 days prior to harvest. The crystallization reagent consisted of 24% polyethylene glycol 8000, 0.167 M calcium acetate, 0.1 M MES pH 6.17. Glycerol was added to a final concentration of 20% (v/v) as a cryoprotectant. Initial screening for diffraction was carried out using the Stanford Automated Mounting system (SAM) [13] at the Stanford Synchrotron Radiation Lightsource (SSRL, Menlo Park, CA). Data were collected at 3 wavelengths corresponding to the inflection(l_1_), high remote(l_2_) and peak energy(l_3_) of a selenium MAD (multi-wavelength anomalous dispersion) experiment at 100 K using a MARCCD 325 detector (Rayonix) at Stanford Synchrotron Radiation Lightsource (SSRL) beamline 9_2. Data processing was carried out using XDS [14] and the statistics are presented in Table 1. The structure was determined by the MAD method using programs SHELX [15] and autoSHARP [16], and refinement was carried out using REFMAC5 [17]. The structure was validated using the JCSG Quality Control server ( http://smb.slac.stanford.edu/jcsg/QC).

**Table 1:** **Summary of crystal parameters, data collection, and refinement statistics for Acel_2062 from *acidothermus cellulolyticus 11b *** **[PDB:3e11]**

**Data collection**	**λ **_**1 **_**MADSe**	**λ **_**2 **_**MADSe**	**λ **_**3 **_**MADSe**
Wavelength (Å)	0.97949	0.91837	0.97897
Resolution range (Å)	29.437-1.800	29.412-1.801	29.399-1.803
No. of observations	49,068	49,153	48,819
No. of unique reflections	19,523	19,533	19,440
Completeness (%)	95.3 (94.6)^a^	95.5 (93.5)^a^	94.9 (90.2)^a^
Mean *I/σ*(*I*)	8.50 (1.69)^a^	8.31 (1.67)^a^	8.08 (1.51)^a^
*R*_*sym*_ on *I* (%)^†^	6.3 (48.0)^a^	6.4 (45.7)^a^	6.8 (55.5)^a^
*R*_*meas*_ on *I* (%)^‡^	9.1 (63.8)^a^	9.6 (64.7)^a^	10.5 (74.3)^a^
Highest resolution shell (Å)	1.86-1.80	1.86-1.80	1.86-1.80
Model and refinement statistics			
Resolution range (Å)	29.44-1.80		
No. of reflections (total)	19,518		
No. of reflections (test)	1,004		
Completeness (%total)	99.0		
Data set used in refinement	λ _1_ MADSe		
Cutoff criteria	|F| > 0		
*R*_*cryst*_^¶^	0.175		
*R*_*free*_^§^	0.204		
Stereochemical parameters			
Restraints (RMSD observed)			
Bond angle (°)	1.386		
Bond length (Å)	0.014		
Average isotropic *B*-value (Å^2^)	11.273		
ESU^††^ based on R_free_ (Å)	0.125		
Protein residues/atoms	228/1791		
Water/Ions	193		

^a^highest resolution shell.

^†^*R*_*sym*_ = S|I_i_- < I_i_ > |/S|I_i_| where I_i_ is the scaled intensity of the i^th^ measurement and < I_i_ > is the mean intensity for that reflection.

^‡^*R*_*meas*_ is the redundancy-independent *Rsym*. [18,19].

^¶^*R*_*cryst*_ = S| |*F*_obs_|-|*F*_calc_| |/S|*F*_obs_| where *F*_calc_ and *F*_obs_ are the calculated and observed structure factor amplitudes, respectively.

^§^*R*_*free*_ = as for *R*_*cryst*_, but for 4.9% of total reflections chosen at random and omitted from refinement.

^†^This value represents the total B that includes TLS and residual B components.

^‡^ESU = Estimated overall coordinate error [20].

Values in parentheses are for the highest resolution shell. Space group: P12_1_1. Unit cell parameters: a = 39.03 Å b = 58.82 Å c = 46.93 α = γ = 90° β = 93.51.

### Determination of oligomeric state

The oligomeric state of the Acel_2062 protein in solution was determined using a 0.8 × 30 cm^2^ Shodex Protein KW-803 size exclusion column (Thomson Instruments) [9] pre-calibrated with gel filtration standards (Bio-Rad). The mobile phase consisted of 20 mM Tris pH 8.0, 150 mM NaCl, and 0.02% (w/v) sodium azide. The apparent molecular weight was calculated using the Bio-Rad Gel Filtration Standard set (#151-1901) and a linear regression of log10MW.

### Bioinformatics

To find homologues of the Acel_2062 protein, a Blastp search was conducted against the non-redundant protein sequence database at NCBI [21], using standard parameters. Structure diagrams were prepared using PyMol. Domain diagrams were taken from Pfam release 27 [22]. Secondary structure topology diagrams were generated by HERA [23] and downloaded from PDBSum website ( http://www.ebi.ac.uk/pdbsum/). The alignment was prepared using MAFFT [24] and ESPript 2.2 ( http://espript.ibcp.fr/ESPript/cgi-bin/ESPript.cgi). PISA analysis [25] of the dimer interface was performed using the PDBe server at the European Bioinformatics Institute ( http://www.ebi.ac.uk/msd-srv/prot_int/). The electrostatic surface was displayed using PyMol ( http://www.pymol.org/) and a Delphi [26] embedded script kindly provided by Qingping Xu. Coot [27] was used to superimpose structures from the Protein Data Bank (PDB). Molecular graphics and analyses were performed with the UCSF Chimera package [28]. The theoretical pI was calculated using the Expasy website ( http://web.expasy.org/compute_pi).

**Figure 1:**
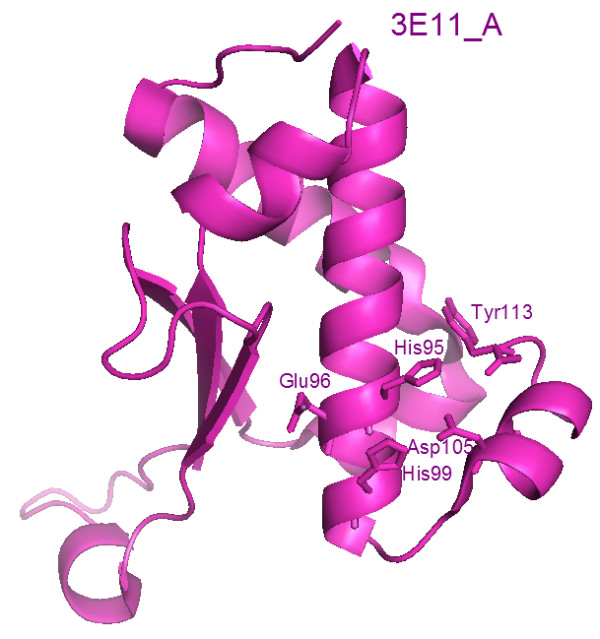
**Crystal structure.** The crystal structure of the Acel_2062 protein from *Acidothermus cellulolyticus*, shown in magenta, reveals a single zincin-like domain. The putative zinc ligands (His95, His99 and Asp105), the putative catalytic Glu96, and Tyr113 are shown as sticks.

## Results and discussion

### Structure description

The crystal structure of the Acel_2062 protein was determined to 1.8 Å resolution using the MAD phasing method. Atomic coordinates and experimental structure factors to 1.8 Å resolution [PDB:3e11] have been deposited in the Protein Data Bank ( http://www.wwpdb.org, [29]). Data-collection, model and refinement statistics are summarized in Table 1. The final model includes two protein molecules (residues 1–113), two acetate molecules, two (presumed to be structural) calcium ions and 193 water molecules in the asymmetric unit. The calcium ions are near the centre of the dimer interface, and may be important for dimerization. The calcium ions are coordinated by Asp18, Glu38 and via waters by Glu15. No zinc was found in the structure, either because little zinc was present during purification and crystallization, the enzyme is in latent state or the protein is not an enzyme. The Matthews coefficient (*V*_*M*_; [30]) is 2.07 Å^3^/Da and the estimated solvent content is 40.54%. The Ramachandran plot produced by *MolProbity*[31] shows that 98.0% of the residues are in favoured regions, with no outliers. The side-chain atoms of Glu22, Asp37, Glu43 and Glu106 on chain A and Glu15, Glu43 and Glu110 on chain B had poor electron density and were omitted from the model. For monomer A, the structure is composed of four helices, two 3_10_ helices and three beta strands (see Figure 1). In monomer B, the N-terminus forms a fourth strand and the dimer is formed from this strand inserting into the beta sheet of monomer A.

The Acel_2062 protein was crystallized as a dimer, with the putative active sites at opposite ends of the dimer. PISA analysis of the structure indicates that the solvent-excluded surface area for the proposed dimer is ~639 Å^2^. From size exclusion chromatography, the molecular weight of the Acel_2062 protein in solution is estimated to be 26,824, which is a ratio of 2.09 over the expected molecular weight of the monomer (12,833) indicating that the protein exists as a dimer (Additional file 1: Figure S1).

The crystal structure of Acel_2062 protein has a minimal zincin-like fold because it retains a three-stranded mixed beta-sheet (rather than the five-stranded beta-sheet of many zincins), the loops are much shorter and the overall sequence length of all members of this family (~110 aa) is significantly shorter than the average for a matrix metalloprotease (MMP)-like domain (~160 aa). The distant homology prediction program FFAS [32] recognizes this similarity, with a marginal statistical significance (Z-score of −8.9 as compared to −9.5 as the significant threshold), suggesting that DUF1025 family is distantly related to metalloproteases.

There is no signal peptide, and the Acel_2062 protein is presumably intracellular.

### Putative active site

**Figure 2:**
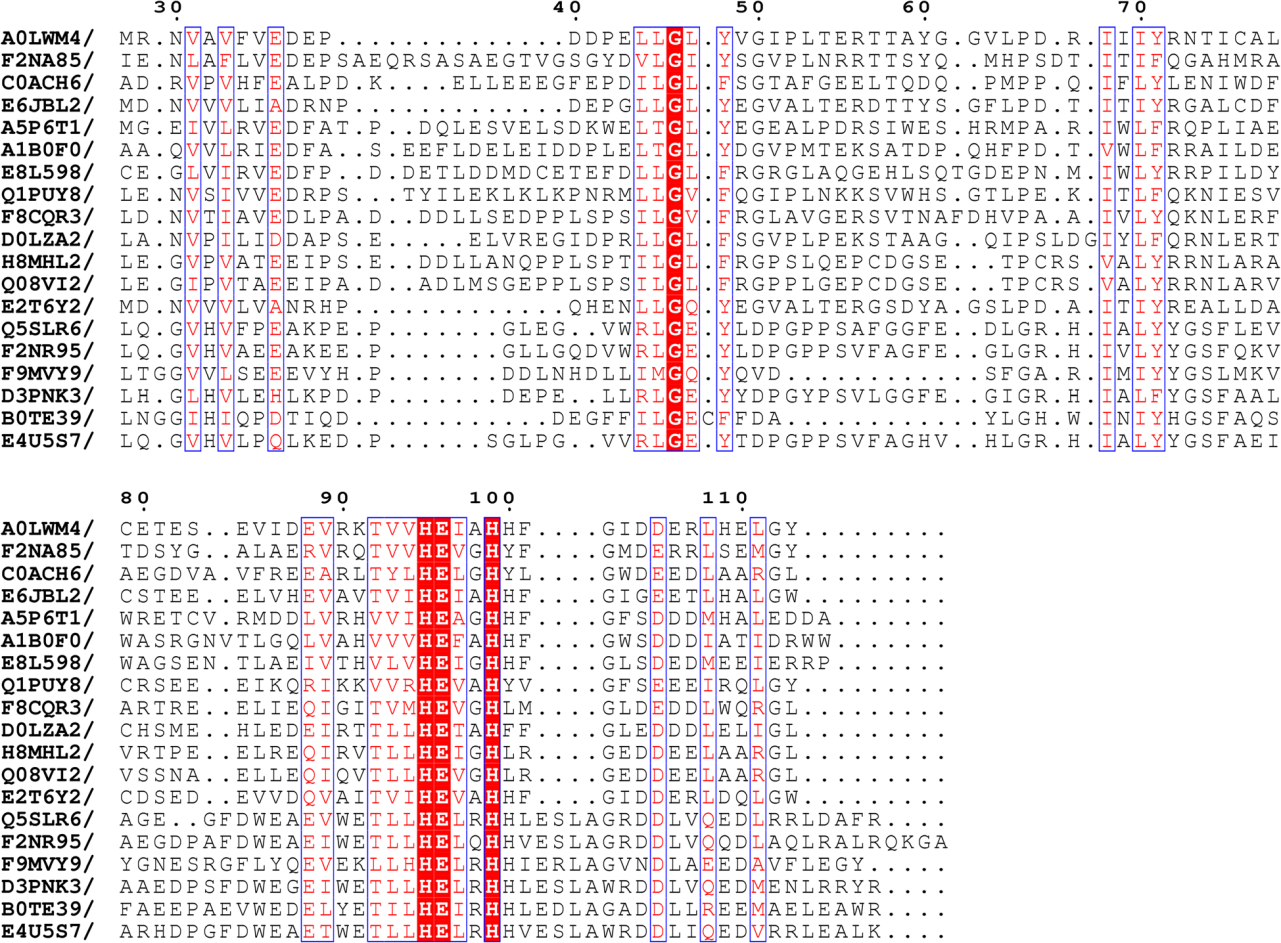
**Sequence alignment of the Acel_2062 protein and a selection of its homologues.** The UniProt accession and the range of the peptidase domain are shown on the left. The zincin motif is boxed in red. Conserved residues shown in white text highlighted in red. Key to sequences (ordered locus name, species): A0LWM4[UniProt:A0LWM4] (Acel_2062, *Acidothermus cellulolyticus*), F2NA85[UniProt:F2NA85] (Corgl_0144, *Coriobacterium glomerans*), C0ACH6[UniProt:C0ACH6] (ObacDRAFT_6101, *Diplosphaera colitermitum*), E6JBL2[UniProt:E6JBL2] (ES5_13138, *Dietzia cinnamea*), A5P6T1[UniProt:A5P6T1] (ED21_26213, *Erythrobacter sp. SD-21*), A1B0F0[UniProt:A1B0F0] (Pden_0883, *Paracoccus denitrificans*), E8L598[UniProt:E8L598] (Met49242DRAFT_2641, *Methylocystis* sp. ATCC 49242), Q1PUY8[UniProt:Q1PUY8] (kustc0300, *Candidatus Kuenenia stuttgartiensis*), F8CQR3[UniProt:F8CQR3] (LILAB_30480, *Myxococcus fulvus*), D0LZA2[UniProt:D0LZA2] (Hoch_3865, *Haliangium ochraceu*), H8MHL2[UniProt:H8MHL2] (COCOR_02006, *Corallococcus coralloides*), Q08VI2[UniProt: Q08VI2] (STAUR_2801, *Stigmatella aurantiaca*), E2T6Y2[UniProt:E2T6Y2] (TMBG_02375, *Mycobacterium tuberculosis*), Q5SLR6[UniProt:Q5SLR6] (TTHA0227, *Thermus thermophilus*), F2NR95[UniProt:F2NR95] (Marky_2224, *Marinithermus hydrothermalis*), F9MVY9[UniProt:F9MVY9] (HMPREF9130_1347, *Peptoniphilus* sp. oral taxon 375 str. F0436), D3PNK3[UniProt:D3PNK3] (Mrub_0627, *Meiothermus ruber*), B0TE39[UniProt:B0TE39] (Helmi_16090, *Heliobacterium modesticaldum*), E4U5S7[UniProt:E4U5S7] (Ocepr_0229, *Oceanithermus profundus*).

**Figure 3:**
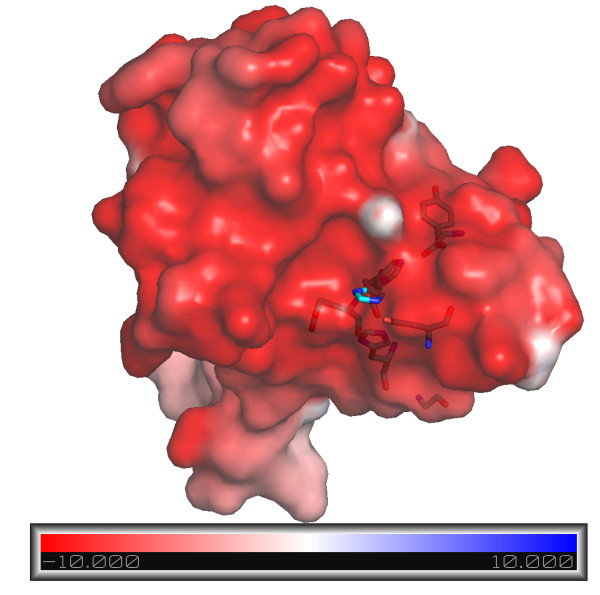
**Electrostatic surface of the Acel_2062 protein.** The electrostatic surface potential representation of the active pocket was made with a Delphi embedded JCSG script, (kindly provided by Qingping Xu) and displayed using Pymol. Negative electrostatic potential predominates over the entire surface of Acel_2062. The putative active site residues (Glu96 and Tyr113) and zinc ligands are (His95, His99 and Asp105) are shown in sticks. The color scale is in units of kT/e ranging from −10 to +10.

From the presence of the HEXXHXXGXXD motif, the potential zinc ligands in the Acel_2062 protein are predicted to be His95, His99 and Asp105, and Glu96 is predicted to be a catalytic residue. In the crystal structure (PDB:3E11), the Glu96 is hydrogen-bonded to five water molecules in both monomers. Conservation of the active site residues is shown in Figure 2. Of the two active sites in both monomers A and B, the one in B is empty and the other in A is occupied by a single water molecule which is coordinated by His95 (at 2.7 Å), His99 (at 3.3 Å) and Asp105 (at 2.9 Å), exactly where the zinc ion would be expected to be. Delphi calculations show that the entire pocket is very acidic. There are five negatively charged residues, plus the carboxyl group from the C-terminus in that area. The surface electrostatic potential is shown in Figure 3. The molecule is negatively charged overall (with 15 Glu and 11 Asp compared to one Lys and eight Arg), with a theoretical pI calculated to be 4.29. This may be an adaptation to the acidic hot spring environment in which *Acidothermus cellulolyticus* lives.

**Figure 4:**
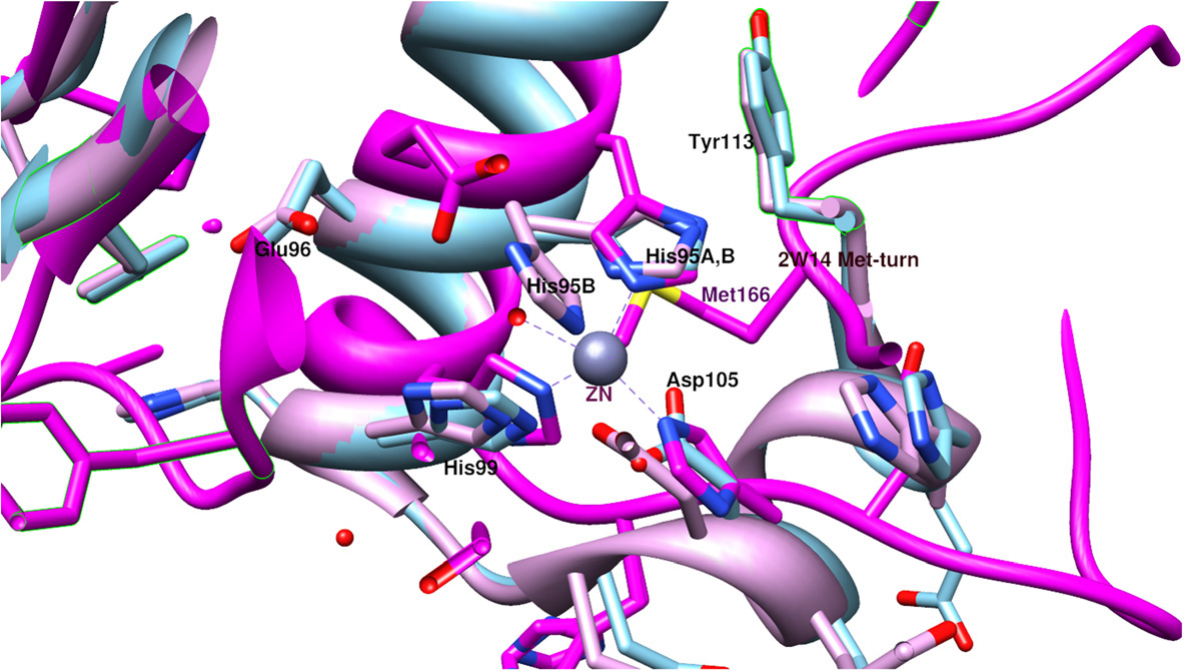
**Superposition of the active sites of the Acel_2062 protein and Bap1 peptidase from *Bothrops asper*.** The comparison between the active centres containing the HEEXH motif; Coot ssm superposition of the two PDB entries (3E11 and 2 W14), has been displayed using Chimera; 3E11-A monomer is shown in turquoise, B in pink, 2 W14 is shown in magenta.

In the Acel_2062 protein, and other members of the family, the HEXXH motif is very close to the C-terminus. Like Glu- and Asp-zincins, there is no Met-turn. The first His (His95) of the HEXXH motif exists in two conformations in monomer B. In the more stable conformation, His95 is electrostatically bound to the carboxyl group of the C-terminal residue (Tyr113). This is the situation also found in monomer A, so it is possible that the water (and presumably also the zinc) only bind when His95 and Tyr113 interact. The structure of the Acel_2062 protein was superimposed upon that of the reprolysin BaP1 peptidase from the snake *Bothrops asper* [PDB:2w14] and this clearly shows that Tyr113 occupies a similar position to the methionine of the Met-turn in the BaP1 peptidase (see Figure 4). So although there is no Met-turn, Tyr113 may compensate for it.

### Sequence similarities

Over 500 homologues of the Acel_2062 protein were found from the Blastp search. In 80 of these proteins, the HEXXHXXGXXD motif is not conserved. The third zinc ligand has been replaced in many of these homologues, often with glutamic acid, which is the third zinc ligand in Glu-zincins.

All of the homologues are from bacteria belonging to seven different phyla. Most homologues come from species in the phylum Firmicutes (294), which are Gram-positive bacteria. There are 185 homologues from species in the phylum Proteobacteria, fourteen from Chloroflexi, five from Planctomycetes, three from Verrucomicrobia, and one each from species in the phyla Caldiserica and Nitrospirae. Most species have only one homologue, but *Stigmatella aurantiaca* has two, though only one has the third zinc ligand conserved.

**Figure 5:**
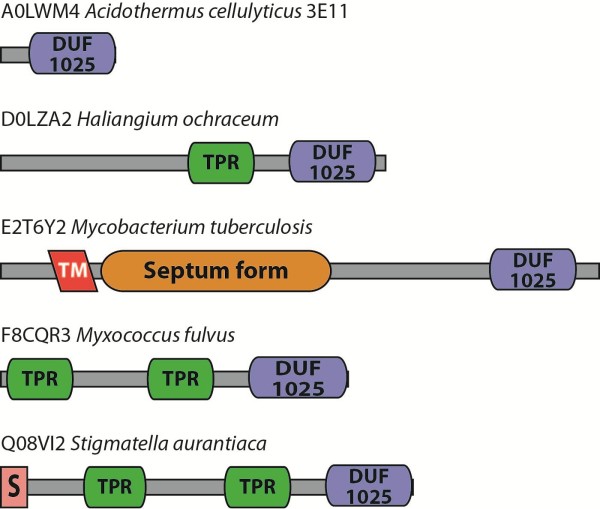
**Domain architectures for the Acel_2062 protein and its homologues.** The different domain architectures for proteins containing an M94 metallopeptidase domain is shown. The UniProt identifier and source organism are given for an example of each domain architecture. Key to domains: DUF1025 is the metallopeptidase domain;TPR, tetratricopeptide repeats; TM, transmembrane region; Septum form, a domain found in proteins predicted to play a role in septum formation during cell division; S, signal peptide.

The different Pfam domain architectures for members of this family are shown in Figure 5. The vast majority of homologues have the simple domain architecture of the Acel_2062 protein. Seventeen homologues include tetratricopeptide repeats (TPR), which mediate protein-protein interactions and the assembly of multiprotein complexes [33]. A TPR repeat motif consists of several tandem repeats of a 34-residue sequence. Eight proteins with TPR repeats are predicted to have signal peptides and are presumably secreted. A homologue from *Mycobacterium tuberculosis* possesses an N-terminal transmembrane domain and a domain found in proteins known to be important for septum formation during spore formation [34].

### Structural similarities

Amongst known metallopeptidases, DALI analysis shows that the Acel_2062 protein structure is most similar to that of a Met-zincin from the archaean *Methanocorpusculum labreanum* (peptidase family M54; archaelysins or archaemetzincins [PDB:3lmc]). The Acel_2062 protein structure is also similar to the carboxy-terminal domain (residues 511 to 624) of *Escherischia coli* HtpG/Hsp90 protein, which is a chaperone protein. This C-terminal domain is important for dimerization, but the mechanism of dimerization via the C-terminal helices is completely different to that of the Acel_2062 dimer. Two of the helices and the beta sheet can be superimposed, and the beta strands run in the same direction. The relationship between Hsp90 and a zincin has not previously been recognized in either the SCOP [35] or CATH [36] databases, and suggests a common evolutionary origin. These structural relationships cover the entire Acel_2062 protein sequence.

The Met-turn is important in all Met-zincins because the methionine is crucial for structurally stabilizing the active site. Even in snapalysins from *Streptomyces*, which also have short sequences, there is a conserved methionine approximately ten residues C-terminal to the third zinc ligand. In the Acel_2062 protein although there are no residues that correspond to the C-terminal 36 residues of the archaemetzincin, which includes the essential Met168, the C-terminal Tyr113 occupies a similar position to the methionine. Although it is tempting to suggest that this tyrosine performs a similar role, it should be noted that the tyrosine is present in only 75 homologues in the family and replaced by tryptophan in a further 220 homologues and by phenylalanine in a homologue from *Rhodococcus* sp. AW25M09. In a further 221 homologues, the full-length sequence falls short of Tyr113. In astacin, a tyrosine (Tyr149) has been shown to be an important residue, contributing to transition state binding, which can be replaced with much reduced efficiency by phenylalanine [37]. If proven to be a peptidase, the Acel_2062 protein and its homologues would form family M94 in MEROPS.

**Figure 6:**
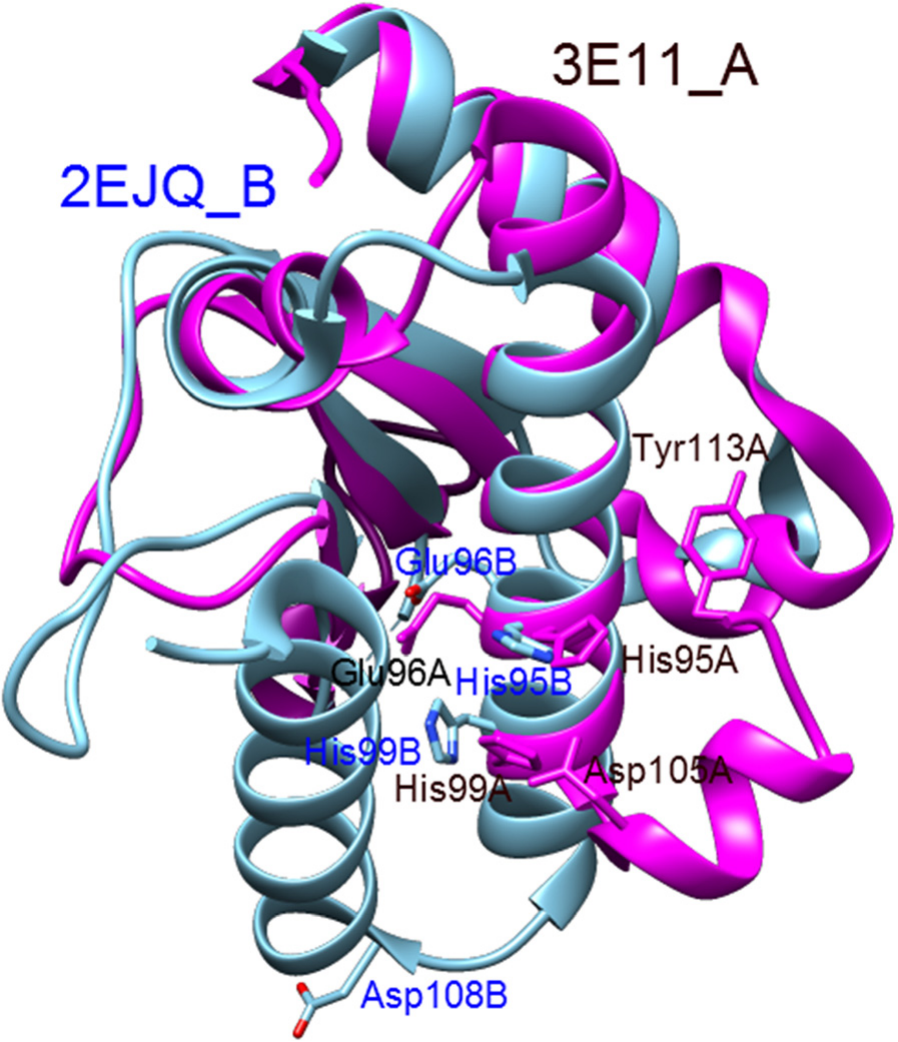
**Superposition of structures for the Acel_2062 and TTHA0227 proteins.** The structure for the Acel_2062 protein (chain A from PDB entry 3E11) is shown in magenta, and the structure for the TTHA0227 protein (chain B from 2EJQ) is shown in cyan. The putative zinc ligands (His95, His99, Asp105 and Asp108) and active site residues (Glu96 and Tyr113) are shown as sticks. Note that the terminal helices do not superimpose, and that Asp108 from the 2EJQ structure is not interacting with the other putative zinc ligands.

The structure of another putative metallopeptidase, the TTHA0227 protein from *Thermus thermophilus* ([PDB:2ejq]; unpublished), is similar. The TTHA0227 protein is also a dimer but has been crystallized with a single magnesium ion, and the structure also lacks any zinc ions. This is also an acidic protein with a theoretical pI calculated to be 4.53. The TTHA0227 protein also contains the Asp-zincin metal-binding motif, and the potential zinc coordinating residues are His95, His99 and Asp109. Chain A is shorter at the C-terminus, lacking residues 109–130, which means that the third zinc ligand is missing. The putative catalytic residue is Glu96. There is a four-residue insert preceding the essential glycine (Gly106, Additional file 2: Figure S2). In chain A, the final helix is continuous, whereas in chain B, there is a turn and there are two, opposing helices. This second helix does not superimpose with the final helix in the 3E11 structure, because it is pointing in the opposite direction so that the faces of the helices that oppose each other in 2EJQ are different from those in 3E11 (Figure 6).

**Figure 7:**
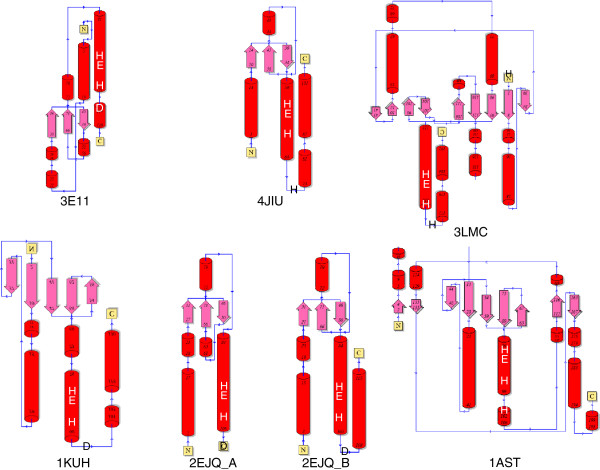
**Secondary structure topologies for the Acel_2062 protein and other metallopeptidases.** Helices are shown as red barrels, strands are shown as pink arrows and connecting regions of random coil and turns are shown as thin blue arrows. “N” and “C” indicate the N- and C-terminus. Numbers indicate the residue range of each structural element. The positions of the zinc ligands and catalytic Glu are indicated by the letters “H”, “D” and “E”. Some images have been rotated and/or flipped to that these active site residues are in equivalent positions. Key to structures: 3E11[PDB:3e11], mini- zincin, *Acidothermus cellulolyticus*; 4JIU[PDB:4jiu], mini-Glu-zincin, *Pyrococcus abyssi*; 3LMC[PDB:3lmc], archaelysin, *Methanocorpusculum labreanum*; 1KUH[PDB:1kuh], snapalysin, *Streptomyces caespitosus*; 2EJQ[PDB:2ejq], mini- zincin, *Thermus thermophilus*; 1AST[PDB:1ast], astacin, *Astacus astacus*.

There are several orthologues of the TTHA0227 protein which have the insert within the Asp-zincin motif. In the homologues from *Oceanithermus profundus* and *Meiothermus ruber* the glycine (Gly106), which in Met-zincins is important for the turn that permits the zinc ligands to face one another, is replaced by tryptophan. It had been thought that only a glycine was acceptable in this position [38], although several bacterial homologues of pappalysin, family M43, have asparagine at this position, including a homologue from *Methanosarcina acetivorans* for which the crystal structure has been solved [39], and a homologue from *Cytophaga hutchinsonii* (Chut1718 gene product) has threonine at this position. Comparisons of the structural topologies of various Met-zincins and the mini-Glu-zincin are shown in Figure 7.

The structure of the Acel_2062 protein represents the minimum sequence known for a zincin domain. The question remains: is this a situation that has developed within this family, or is it a relic of the ancestral zincin gene? One way to answer this question is to look at the species distribution of peptidases from the various families in the clan. Table 2 shows the phyletic distribution of all families within clan MA. The number of phyla within each of the three superkingdoms (Archaea, Bacteria, Eukaryota) containing at least one homologue within each family is shown. Amongst the Glu-zincins, families M1, M3, M32 and M48 are widely distributed in phyla from all three superkingdoms (M41 is also widely distributed in bacteria and eukaryotes, but is absent from archaea). The mini-Glu-zincins, from family M95, have a much narrower distribution and are absent from eukaryotes. These observations imply that the last common universal ancestor most likely possessed a homologue from each of these families, and that the larger Glu-zincin structure is the ancestral state. The distribution of Asp- and Met-zincins is more restricted in all families, and the only family that is well represented in all domains of life is family M54, the archaelysins. There are homologues from archaea (93 species), bacteria (47 species) and eukaryotes (69 species), though these are found in less than half of the bacterial and eukaryote phyla. It is likely that an archaelysin most closely represents the ancestral Met-zincin structure. Archaelysin possesses the Met-turn [40,41], so the implication is that the Met-turn has been lost from an ancestor of family M94 and functionally replaced by a C-terminal aromatic residue (tyrosine or phenylalanine). The much narrower distribution of members of M94 supports the hypothesis that the family is a more recent development.

**Table 2:** Phyletic distribution of experimentally confirmed and hypothetical peptidase families in MEROPS clan MA

***Subclan***	***Family***	***Name***	***Archaea***	***Bacteria***	***Eukaryota***
***Total phyla***			5	28	49
MA(E)	M1	Aminopeptidase N	**3**	**21**	**31**
	M2	Angiotensin-converting enzyme	-	6	11
	M3	Thimet oligopeptidase	**3**	**25**	**28**
	M4	Thermolysin	1	10	4
	M5	Mycolysin	-	2	-
	M9	Microbial collagenase	1	6	-
	M13	Neprilysin	1	12	23
	M26	IgA metalloendopeptidase	1	4	-
	M27	Tentoxilysin	-	1	-
	M30	Hyicolysin	1	9	-
	M32	Carboxypeptidase Taq	***5***	**16**	**7**
	M34	Anthrax lethal factor	-	1	-
	M36	Fungalysin	-	6	5
	M41	FtsH endopeptidase	-	***27***	***36***
	*M47*	*Metallopeptidase PRSM1*	*-*	*-*	*1*
	M48	Metallopeptidase STE24	**4**	**25**	**28**
	M49	Dipeptidyl-peptidase III	-	6	21
	M56	BlaR1 peptidase	-	11	-
	M60	Enhancin	-	3	1
	M61	Glycyl aminopeptidase	2	10	2
	*M65*	*YugP protein (Bacillus)*	*-*	*3*	*-*
	*M69*	*F19C6.4 protein (Caenorhabditis)*	*-*	*-*	*1*
	*M70*	*Surface protein (Ehrlichia)*	*-*	*1*	*-*
	M76	Atp23 peptidase	-	-	21
	M78	ImmA peptidase	-	10	-
	M85	NleC peptidase	-	1	-
	M90	MtfA peptidase	-	9	-
	M91	NleD peptidase	-	1	-
	*M93*	*BACCAC_01431 protein (Bacteroides)*	*-*	*1*	*-*
	M95	Proabylysin	1	5	-
MA(M)	M6	Immune inhibitor A	1	***12***	3
	M7	Snapalysin	-	1	-
	M8	Leishmanolysin	-	7	20
	M10	Matrix metallopeptidases	2	11	14
	M11	Autolysin	-	2	2
	M12	Astacins/reprolysins	1	8	***26***
	M35	Deuterolysin	-	3	3
	*M39*	*YIL108W protein (Saccharomyces)*	*-*	*-*	*1*
	M43	Cytophagalysin	1	6	14
	M54	Archaelysin	***4***	***12***	8
	M57	PrtB protein (*Myxococcus*)	-	3	-
	*M59*	*Putative zinc metalloprotease (Vibrio)*	*-*	*1*	*-*
	*M62*	*Membrane metalloprotease (Euryarchaeota)*	*1*	*3*	*-*
	M64	IgA peptidase (*Clostridium*)	-	5	1
	M66	StcE peptidase	-	3	1
	*M68*	*JHP0742 protein (Helicobacter)*	*1*	*3*	*-*
	*M71*	*PAE0478 protein (Pyrobaculum)*	*1*	*-*	*-*
	M72	Peptidyl-Asp metallopeptidase	-	7	1
	M80	Wss1 peptidase	-	-	12
	*M83*	*DR2310 peptidase*	*-*	*2*	*-*
	M84	mpriBi peptidase	1	1	-
	*M94*	*Acel_2062 protein (Acidothermus)*	*--*	*9*	*-*

Families are ordered by subclan and name. The number of phyla in each of the superkingdoms Archaea, Bacteria and Eukarya from which at least one homologue is known are shown. Where the number of phyla exceeds half the known phyla in the superkingdom, the number is highlighted in bold. The family in each subclan with examples from the most phyla in each superkingdom is shown as bold, italics text. A hyphen indicates no examples are known in that superkingdom. Rows in italics are families of putative peptidases that have not yet been experimentally characterized as such. The MEROPS identifier for such a family is provisional and may be subject to change.

## Conclusions

The Acel_2062 protein from *Acidothermus cellulolyticus* is a protein of unknown function, but was predicted to be a metallopeptidase from the presence of a motif (**H**EXX**H**XXGXX**D**) conserved amongst the Asp-zincins, which contain a single, catalytic zinc ion ligated by the histidines and aspartic acid within the motif. The tertiary structure of the Acel_2062 protein was determined by the Joint Center for Structural Genomics, and confirmed the presence of a single, zincin-like metallopeptidase-like domain. In our crystallographic model there are two molecules in the asymmetric unit and from size-exclusion chromatography, the protein dimerizes in solution. A water molecule is present in the putative zinc-binding site in one monomer, which is replaced by one of two observed conformations of His95 in the other. The C-terminal Tyr113 may be important for stabilizing the putative active site. Additional experimentation would be required to prove that the Acel_2062 protein is a metallopeptidase.

Although the Acel_2062 protein is structurally related to the zincins, it contains the minimum structural features of a member of this protein superfamily, and can be described as a “mini- zincin”. There is a striking parallel with the structure of a mini-Glu-zincin, which represents the minimum structure of a Glu-zincin (a metallopeptidase in which the third zinc ligand is a glutamic acid). Rather than being an ancestral state, phylogenetic analysis suggests that the mini-zincins are derived from larger proteins.

## Competing interests

The authors declare that they have no competing interests.

## Authors’ contributions

CBT performed X-ray structure determination and prepared some of the figures; NDR, YC, HLA, RYE, PC and MP analysed the sequence-structure-function relationships and prepared the manuscript, tables and the other figures. All authors read and approved the final manuscript.

## Supplementary Material

Additional file 1: Figure S1. Molecular weight determination by size-exclusion chromatography. A). Elution profile for Bio-Rad Gel Filtration Standard set (#151-1901) comprising vitamin B_12_ (1,350 Da), horse myoglobin (17 kDa), chicken ovalbumin (44 kDa), bovine gamma-globulin (158 kDa) and bovine thyroglobulin (670 kDa). B) Elution profile for the Acel_2062 protein. Click here for file

Additional file 2: Figure S2. Crystal structure of the TTHA0227 protein from *Thermus thermophilus.* The PDB entry 2EJQ[PDB:2ejq] is a dimer, and because different elements are missing from both monomers, the structure of both monomers is shown. Chain A is shown in beige, chain B in cyan. Residues described below and in the text are labelled and shown as sticks. The monomers differ in terms of the residues that cannot be resolved. Chain A lacks the C-terminal residues Asp109-Gly130, and chain B lacks residues Pro54-Leu64 as well as the C-terminal Gly-Glu-Gly residues. Also in 2EJQ, Asp109 is too far away from the other potential zinc ligands to be a ligand itself. There are other potential zinc ligands in residues 110–130, including Glu113, Asp114, and Asp119. Only Asp119 is close enough to the imidazolium rings of the histidines to act as the third zinc ligand. Unfortunately, this Asp119 is poorly conserved, whereas Asp109 is well conserved. Because Asp109 and the final helix are close to the dimer interface, the structure here may be distorted because of the dimerization. Click here for file
