# Supplementary figures and images for: New mini- zincin structures provide a minimal scaffold for members of this metallopeptidase superfamily
Source: BMC Bioinformatics. 2014 Jan 3;15:1. doi: 10.1186/1471-2105-15-1 (PMC3890501; doi:10.1186/1471-2105-15-1)

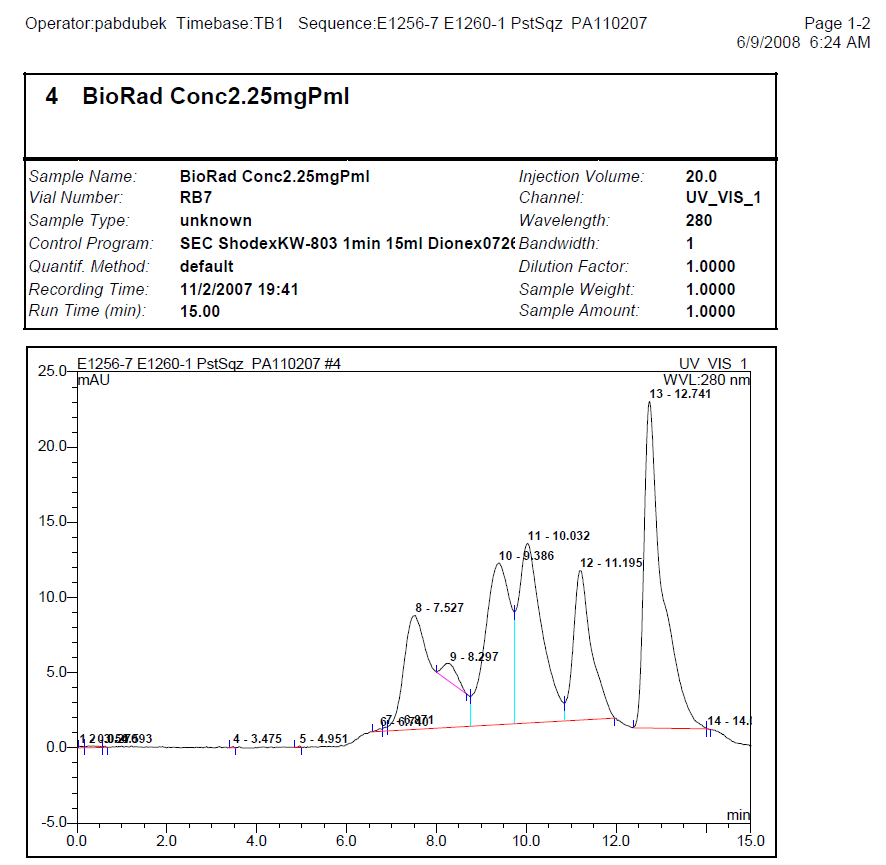

Supplement: Additional file 1: Figure S1. — Molecular weight determination by size-exclusion chromatography. A). Elution profile for Bio-Rad Gel Filtration Standard set (#151-1901) comprising vitamin B12 (1,350 Da), horse myoglobin (17 kDa), chicken ovalbumin (44 kDa), bovine gamma-globulin (158 kDa) and bovine thyroglobulin (670 kDa). B) Elution profile for the Acel_2062 protein. [file 1471-2105-15-1-S1.png]

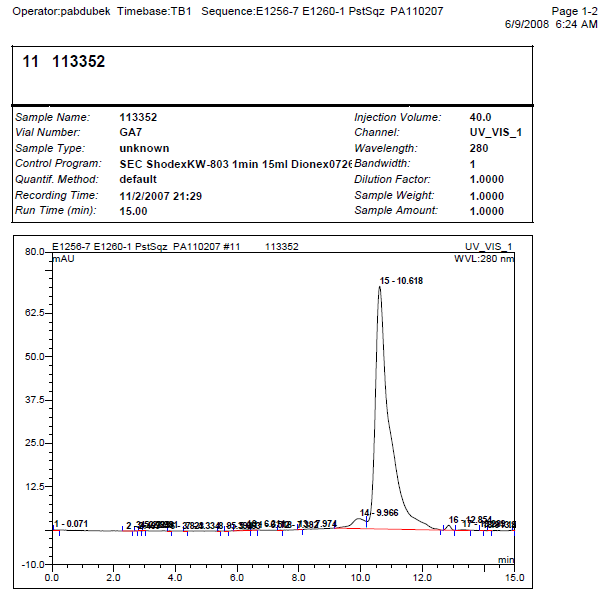

Supplement: Additional file 2: Figure S2. — Crystal structure of the TTHA0227 protein from Thermus thermophilus. The PDB entry 2EJQ[PDB:2ejq] is a dimer, and because different elements are missing from both monomers, the structure of both monomers is shown. Chain A is shown in beige, chain B in cyan. Residues described below and in the text are labelled and shown as sticks. The monomers differ in terms of the residues that cannot be resolved. Chain A lacks the C-terminal residues Asp109-Gly130, and chain B lacks residues Pro54-Leu64 as well as the C-terminal Gly-Glu-Gly residues. Also in 2EJQ, Asp109 is too far away from the other potential zinc ligands to be a ligand itself. There are other potential zinc ligands in residues 110–130, including Glu113, Asp114, and Asp119. Only Asp119 is close enough to the imidazolium rings of the histidines to act as the third zinc ligand. Unfortunately, this Asp119 is poorly conserved, whereas Asp109 is well conserved. Because Asp109 and the final helix are close to the dimer interface, the structure here may be distorted because of the dimerization. [file 1471-2105-15-1-S2.png]
